# Tensile behaviors of layer-to-layer 2.5D angle-interlock woven composites with/without a center hole at various temperatures

**DOI:** 10.1038/s41598-020-71481-1

**Published:** 2020-09-18

**Authors:** Jian Song, Weidong Wen, Haitao Cui, Lixiao Li, Yang Lu

**Affiliations:** 1 Guangdong Provincial Key Laboratory of Durability for Marine Civil Engineering, College of Civil and Transportation Engineering, Shenzhen University, Shenzhen, 518060 China; 2 College of Energy and Power Engineering, Nanjing University of Aeronautics and Astronautics, Jiangsu, 210016 China; 3 Nano-Manufactruing Laboratory (NML), Shenzhen Research Institute of City University of Hong Kong, Shenzhen, 518057 China

**Keywords:** Composites, Mechanical engineering

## Abstract

The temperature-dependent mechanical behaviors of open-hole composite plates are essential for composite design and structures. Here, tensile experiments of shallow straight-link-shaped 2.5D woven composites (abbr. 2.5DWC) with/without a center hole are first conducted at different temperatures (20 °C, 180 °C and 240 °C). Failure modes are examined by scanning electron microscope (SEM). Thermal property of QY8911-IV resin is investigated by DMA analysis. It is noted for samples without the center hole that with the increase of temperature, the tensile stress–strain curves exhibit a linear response until that a slight nonlinearity at the end stage. The strength retention rates at 180 °C and 240 °C are totally equal. For the open-hole samples, it is interestingly found that the strength retention rates are higher than that of samples without the hole at 180 °C, resulting from the stress concentration accommodation and fiber-dominated failure mode. Even at 240 °C, there is no necking phenomenon for all the failed samples, but more broom-like damage extent is observed in the cross-section. Due to the primary load-bearing warp yarns and hole-edge stress concentration, obvious pull-out warp yarns emerge near the hole edge.

## Introduction

The application of carbon fiber reinforced resin matrix woven composites has significantly accelerated in the past few years^1–4^. As a new category of lightweight woven composites, layer-to-layer 2.5D angle-interlock woven composites have a potential application in the field of aerospace engineering such as fan blade and aero-engine case due to the advantages over the conventional laminated composites, including through-thickness reinforcement, high damage tolerance and advanced anti-fatigue characteristic^5,6^. However, some composite structures expose to long-term temperatures in 100–200 °C such as aero-engine casing, which requires that resin matrix composites have a temperature-resistant capacity^7–11^.

Many scholars have reported the mechanical behaviors of laminated composites at room and elevated temperatures^12–16^. But few works are focus on the thermo-mechanical behaviors of woven composites^17–21^. Failure mechanisms in off-axis 2D woven composites at room temperature and 205 °C were experimentally studied by Selezneva et al.^9^. Experimental results shown that primary load-bearing fiber yarns were rotated and straightened out just ahead of failure. Montesano et al. found that the resin softening at 225 °C can effectively mitigate the stress concentration in 2D woven composites, thereby resulting in the failure of fiber yarns^8,22^. In addition, Vielle et al. noted that the overstresses were in the hole edge of woven composites at the temperature higher than the *T*_g_, which can be effectively accommodated by the highly ductile of resin^20^. For the 3D woven composites, the thermo-mechanical responses at high temperatures were rarely studied.

Apart from the thermo-mechanical performance, the fact of joining composites to other structures makes it necessary to drill composite components, which inevitably damages both fibers and matrix in the hole-edge domain^23,24^. However, experimental results indicate that hole has a remarkable impact on the crack propagation of damaged zones and the mechanical properties of laminated/2D woven composites^25–29^. As for the resin matrix laminated with a hole characterization, the occurrence of splitting in composites is sensitive to the mechanical properties at room temperature^30^. Furthermore, large-area delamination along with necking phenomenon was found at elevated temperatures, attributed to the softening and failure of resin matrix^31^. As for the 2D resin matrix woven composites, Vieille and Taleb investigated the thermo-mechanical behaviors of 2D thermosetting-based wove composites with a circle hole, and found that the highly ductile behavior at un-ambient temperature is very effective to accommodate the overstresses near the hole^20^. However, the influence of hole on the mechanical behavior of 2.5D resin matrix woven composites is still unambiguous, especially at elevated temperatures, due to the complexity of 3D-net-shaped architecture.

To sum up, the comprehensive effects of hole and temperature may result in accelerating the disastrous failure of composites, but there is no reference about the influence of them on the mechanical properties and failure mechanism of 2.5D resin matrix woven composites with a center hole. Here, a new class of 2.5D angle-interlock resin matrix woven composites with a shallow straight-link architecture has been experimentally investigated. Note: the matrix is a temperature-resistant resin with a high glass transition temperature (~ 252 °C). The mechanical behaviors and failure mechanisms were studied at 20 °C, 180 °C and 240 °C. Moreover, probable damage propagation mechanisms were discussed. We hope this work could facility the further engineering application of 2.5D woven composites, especially in aero-engine areas (woven fan blade and casing).

## Materials and methods

### Materials and architecture

**Figure 1:**
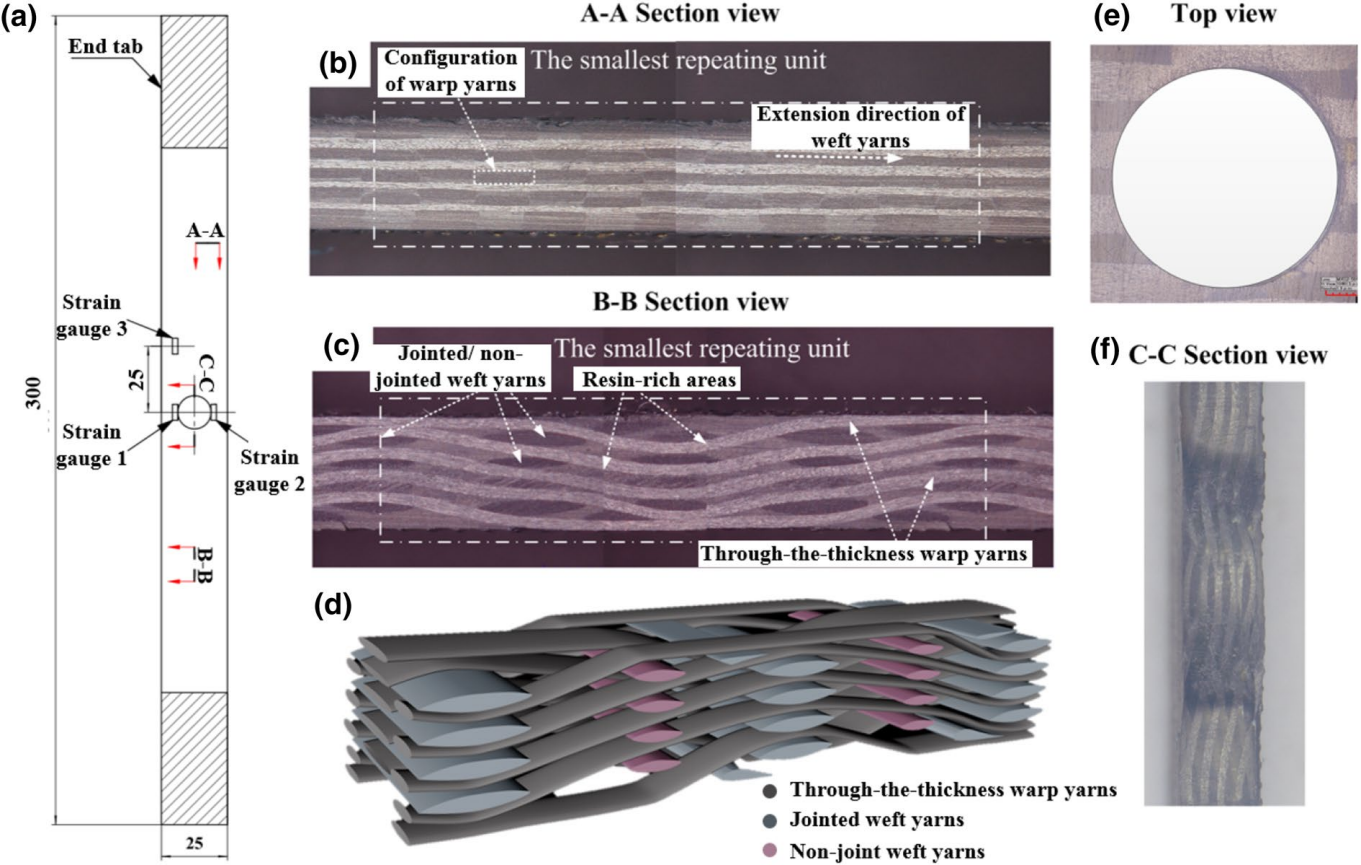
(**a**) Sample geometric sizes. (**b**,**c**) SEM images of architecture of 2.5DWC. (**d**) Schematic illustration of warp yarns and jointed/ non-jointed weft yarns without resin-rich domain. (**e**,**f**) Top and “C–C” section views of the hole-edge domain.

The 2.5D layer-to-layer angle-interlock woven fabric with a shallow straight-link architecture was fabricated using T300 carbon fiber yarns (CF) that consist of 3 K filaments per bundle and the temperature resistance of QY8911-IV resin with a high glass transition temperature 256 °C, indicating that the composites have a great load-bearing capacity at elevated temperatures. A 600 mm × 480 mm flat plate with a fiber volume fraction of ~ 51.62% was fabricated using resin transfer molding (RTM) technology. Afterwards, the plate was subsequently cut into thirty samples with a dimension of 300 × 25 mm^2^ based on abrasive water-jet cutting technology. Drilling operation was carried out using three drill bits with the diameters of 4.1 mm, 6.3 mm and 8.5 mm based on a multi-function drilling machine (BF25-1000W ENYUN™, China). Figure 1a shows the related dimensions of the open-hole samples. In the drilling process, sacrificial back and front plexiglass plates were used to clamp the sample in an effort to mitigate damage in the hole-edge domain, and then the hole edge was lightly blasted using abrasive paper (5,000 mesh) and blown cleanly. In order to avert the breakage in the end, two anvils with a dimension of 50 mm × 25 mm were adhered to the ends of samples. Note: apart from the difference in the hole characteristic, the geometric sizes of samples without a center hole are completely same with those of open-hole ones.

Figure 1b,c illustrates the architecture of 2.5DWC from the warp and weft directions views. It can be seen that the 3D net-shaped fabric was periodically weaved together along the warp and thickness directions. Compared to the shallow curve-link architecture^32^, each warp yarn passes through the adjacent layers of weft yarns in the thickness, but it interlaces the weft yarns with a whole line interval. Thus, in this work, the weft yarns were artificially defined as interlocked weft yarns and non-interlocked weft yarns. Figure 1d plotted a schematic illustration of 2.5DWC without matrix. Such architecture can not only make high tensile strength, but also has a great delamination-resistant capacity. Figure 1e,f exhibits the localized images of hole-edge domain. It can be noted that although the hole edge exists a slight damage induced by drill bit, no obvious delamination phenomenon on the surfaces in the hole-edge domain (Fig. 1e). Apart from the broken yarns cut by drill bit, the 3D net-shaped architecture of fiber yarns still maintains integration (Fig. 1f).

### Experimental method

All the tests were conducted by an MTS 810 hydraulic servo dynamic material test machine. An MTS809 furnace with an integrated temperature controller was used. Test procedure at room temperature referred to the ASTM D 3039^33^, while for the elevated tests, temperature was ramped up at a rate of 10 °C per minute from room temperature to the target temperature in force control, and was allowed to settle for 30 min prior to testing. Meanwhile, a thermocouple was adhered to the specimen surface in advance to ensure that the temperature was the same with the target temperature. Afterwards, the tests were carried out at 20 °C, 180 °C and 240 °C, and the fracture morphologies were inspected using a high-resolution camera (Canon™ EOS-1DX Mark II) and SEM (SEM, Quanta™ 450 FEG).

### Finite element model of open-hole 2.5DWC

In order to analysis the thermo-mechanical stress distribution of open-hole 2.5DWC, finite element analysis (FEA, ANSYS™, 14.5) was adopted. Based on the actual cross-sectional photomicrographs shown in Fig. 1b,c, a new finite element (FE) model of 2.5DWC with a circle hole of ∅4.1 was ultimately established (Corresponding establishment process was given in Fig. S1, Supporting Information).

## Results and discussion

### Effect of temperature on mechanical responses

**Figure 2:**
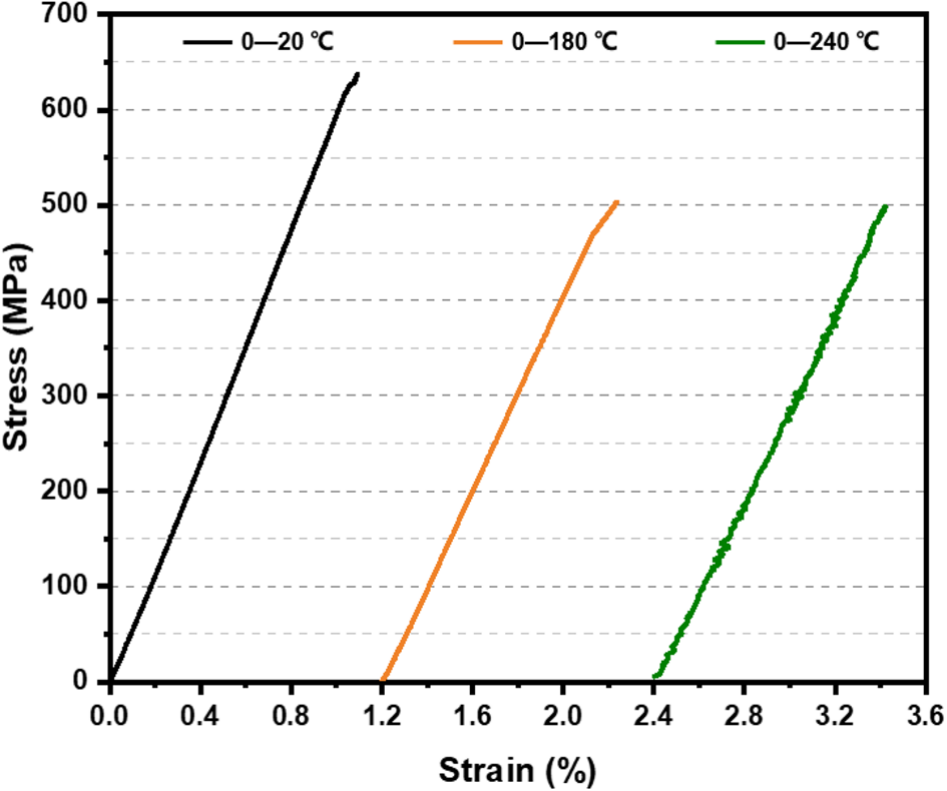
Tensile stress vs. strain curves of 2.5DWC with a center hole at different temperatures.

Figure 2 shows the typical stress vs. strain curves of 2.5DWC without a center hole loaded at 20 °C, 180 °C and 240 °C, and the related strengths as well as hole factor are listed in Table 1. Here, the strength is calculated by dividing the maximum load by the effective load-bearing cross-section. It can be seen from Fig. 2 that the relationship between stress and strain is almost linear for each curve, followed by a slightly nonlinear at the end. Finally, a sudden failure happens, resulting in the loss of load-bearing capacity. It is very similar to that of unidirectional composites loaded along the fiber direction^34,35^.

**Table 1:** Tensile response of open-hole 2.5DWC at different temperatures.

Temperature ( °C)	Hole diameter (mm)	Strength (MPa)	Average strength (MPa)	*C*_h_
20	0	640.26, 651.31, 637.82	643.13	1
	4.1	524.58, 500.29	512.43	0.80
	6.3	453.89, 481.46	467.67	0.73
	8.5	423.30, 427.56	425.43	0.66
180	0	503.08, 469.01, 566.03	512.71	1
	4.1	472.50, 476.17	474.34	0.93
	6.3	447.11, 466.50, 442.35	451.99	0.88
	8.5	392.41, 378.16	385.29	0.75
240	0	508.38, 498.82	503.60	1
	4.1	332.55, 339.47	336.01	0.67
	6.3	329.48, 313.92	321.70	0.64
	8.5	319.47, 296.53	308.00	0.61

*Note* the *C*_h_ represent the hole factor.

The slight nonlinearity of the 2.5DWC without a center hole at 20 °C may be associated with the breakage of load-carrying warp yarns alongside of surrounding matrix failure. The stress–strain curve of the samples with a center hole at 180 °C (*T* < *T*_g_) has a linear behavior at the initial and intermediate parts, followed by a nonlinear response maintaining a very short time. As discussed in Ref.^36^, the stress–strain curve of 2.5DWC (shallow curve-link architecture architecture) at 180 °C had a pronounced nonlinear behavior induced by the softening of matrix at high temperature. As discussed in “Effect of temperature on fracture morphologies of 2.5DWC without a center hole”, the linear stress–strain relationship can be ascribed that the primarily load-bearing component is warp yarn and the average included angle between the warp yarn and loading direction in shallow straight-link 2.5DWC (studied in this work) is less than that in shallow curve-link 2.5DWC. When temperature rises to 240 °C (*T* ~ *T*_g_ = 256 °C), it is meaningful found that although failure resin and resin soften have happened (DMA curve, Fig. 5a), the sample with a center hole retains a relatively high load-bearing capacity and the related stress–strain curve still exhibits a linearity, indicating that the 2.5D composite plate has a great temperature-resistant capacity at the elevated temperature. Such mechanical response could be attributed to the comprehensive effect of temperature and load-carrying warp yarns (Discussed in “Discussion”). Additionally, the sudden failure phenomenon gives a suggestion of fiber-dominated brittle failure mechanism for the 2.5DWC with a center hole.

**Figure 3:**
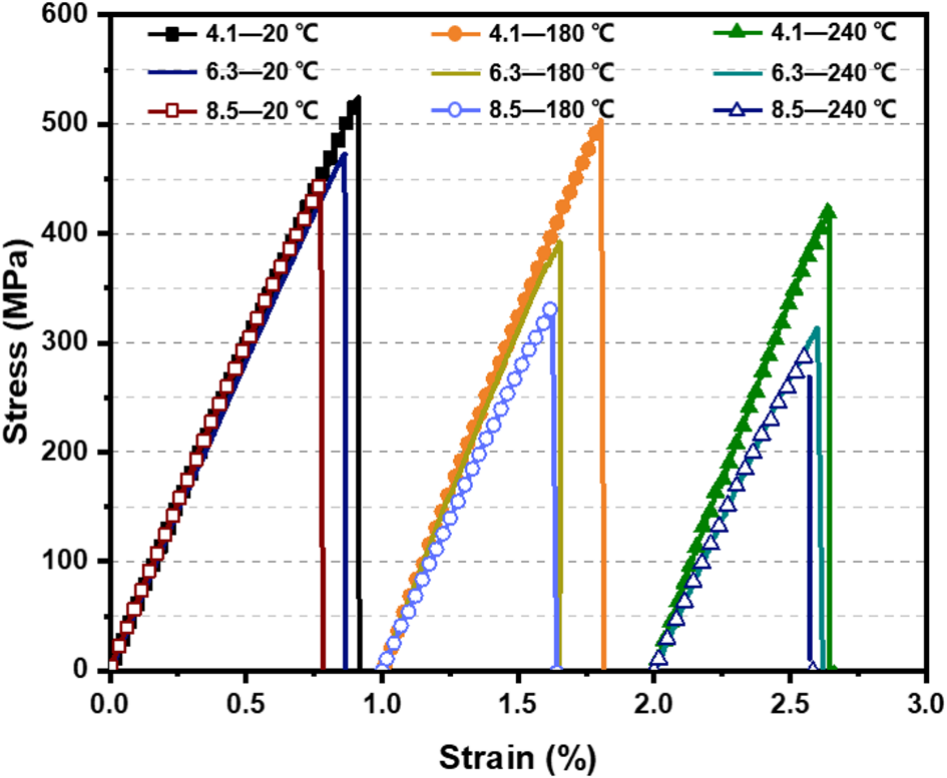
Tensile stress vs. strain curves of open-hole 2.5DWC at different temperatures.

The tensile stress–strain curves of open-hole 2.5DWC at different temperatures are illustrated in Fig. 3, and the strength as well as hole factor are listed in Table 1. As seen in Fig. 3, all the stress–strain curves of open-hole samples are similar, including an initial linear segment, nonlinear segment and sudden failure segment. Compared to the related curve of 2.5DWC without a center hole at the same temperature, the nonlinearity for open-hole 2.5DWC is more obvious, especially at 240 °C, demonstrating the influence of hole on the mechanical responses. It is also interesting to note that the open-hole samples at 180 °C and 240 °C can remain a certain degree of load-carrying and a brittle fracture mode in stress–strain curves also exists. It indicates that the failure mode of open-hole 2.5DWC is still dominated by fiber yarns. For the open-hole samples, a hole factor, *C*_h_, is defined to assess the hole sensitivity of strength, as also listed in Table 1. The hole factor can be obtained as $C_{h} = \left( {\sigma_{u}^{notched} /\sigma_{u}^{unnotched} } \right)_{T}$, where $\sigma_{u}^{notched}$ and $\sigma_{u}^{unnotched}$ mean the strengths of 2.5DWC with and without a center hole tested at the same temperatures. As shown in Table 1, the hole factors of samples at 20 °C and 180 °C are higher than those at 240 °C, resulting from the softening of resin at high temperature, as later confirmed in Fig. 5. This reason was also found in Ref.^20^, where the residual stress at elevated temperature was enhanced compared to that at room temperature. Additionally, when the temperature remains constant, the load-carrying capacity of open-hole 2.5DWC gradually decreases with the increase of hole size, which is consistent with the open-hole laminated composites^29,37^. Nevertheless, the extension of reduction induced by the enlargement of hole size is not significant at 240 °C, indirectly suggesting that the temperature plays a major role in the mechanical response at 240 °C.

**Figure 4:**
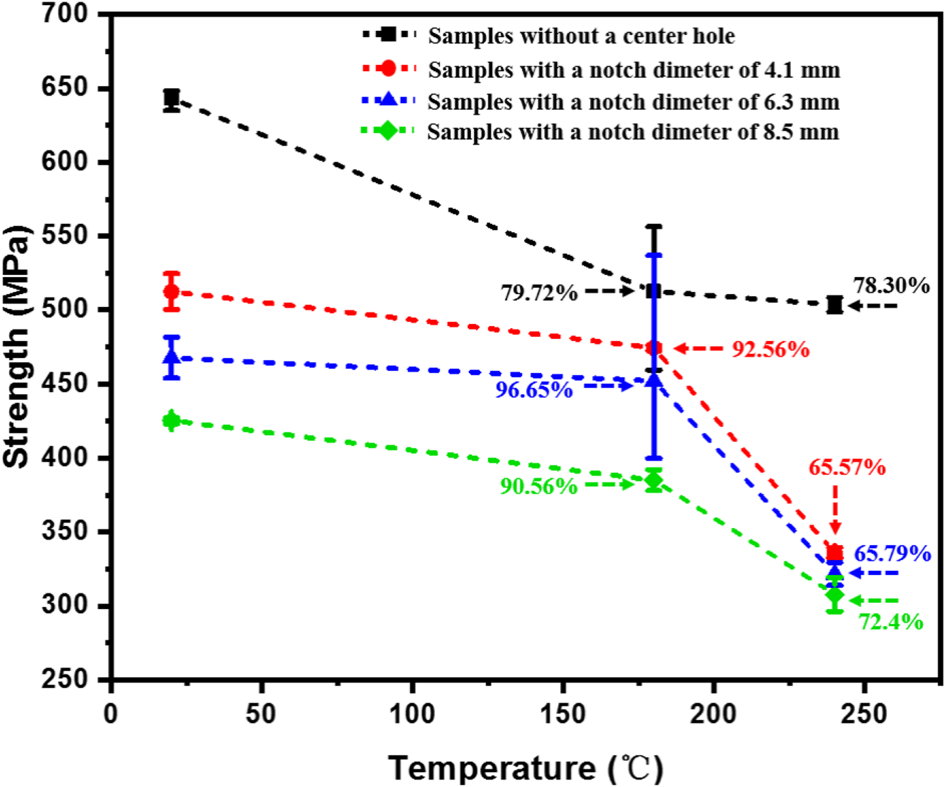
Tensile strength and strength retention rate of 2.5DWC with/ without a center hole as a function of temperature and hole size. Here, the percentage represents the strength retention rate, which can be calculated as $\left. {\sigma_{u}^{{}} } \right|_{T} /\left. {\sigma_{u}^{{}} } \right|_{{T = T_{0} }}$, where $\left. {\sigma_{u}^{{}} } \right|_{{T{ = }T_{0} }}$ and $\left. {\sigma_{u}^{{}} } \right|_{T}$ mean the room and elevated strengths for the samples with the same hole sizes.

The tensile strength of 2.5DWC with/without a center hole as a function of temperature is illustrated in Fig. 4. It can be noted that the strength decreases with the increase of temperature. The strength retention rates of open-hole samples with different hole sizes are obviously higher than that of 2.5DWC without a center hole when the temperature is lower than 180 °C, which can be ascribed to the principal load-bearing weft yarns and stress concentration accommodation. When the temperature rises to 240 °C, it is worth noting that the strength retention rates of samples without a center hole have a slight decline trend, indicating that the 2.5DWC without a center hole remains a high load-carrying capacity at 240 °C. The main reason is that although the mechanical performance of resin is significantly affected by the high temperature (closed to the *T*_g_), the 3D net-shaped carbon fiber yarns (Fig. 1b–d) still maintain a certain load-carrying capacity. For the open-hole samples, the retention rates of open-hole samples at 240 °C experience a relatively large reduction, but the sample with a hole size of 8.5 mm still maintains a 65.79% retention rate, which is higher than the CF/Epoxy angle-ply woven composites studied in Ref.^20^. As mentioned above, the high tensile retention of CF/QY8911-IV 2.5D woven composites can be ascribed to the temperature-resistant characteristic of QY8911-IV resin, load-bearing warp yarns and stress concentration accommodation. The coupling effect of temperature and hole size can be quantitively expressed using Eq. (R1), *SI*.

### Effect of temperature on resin matrix

**Figure 5:**
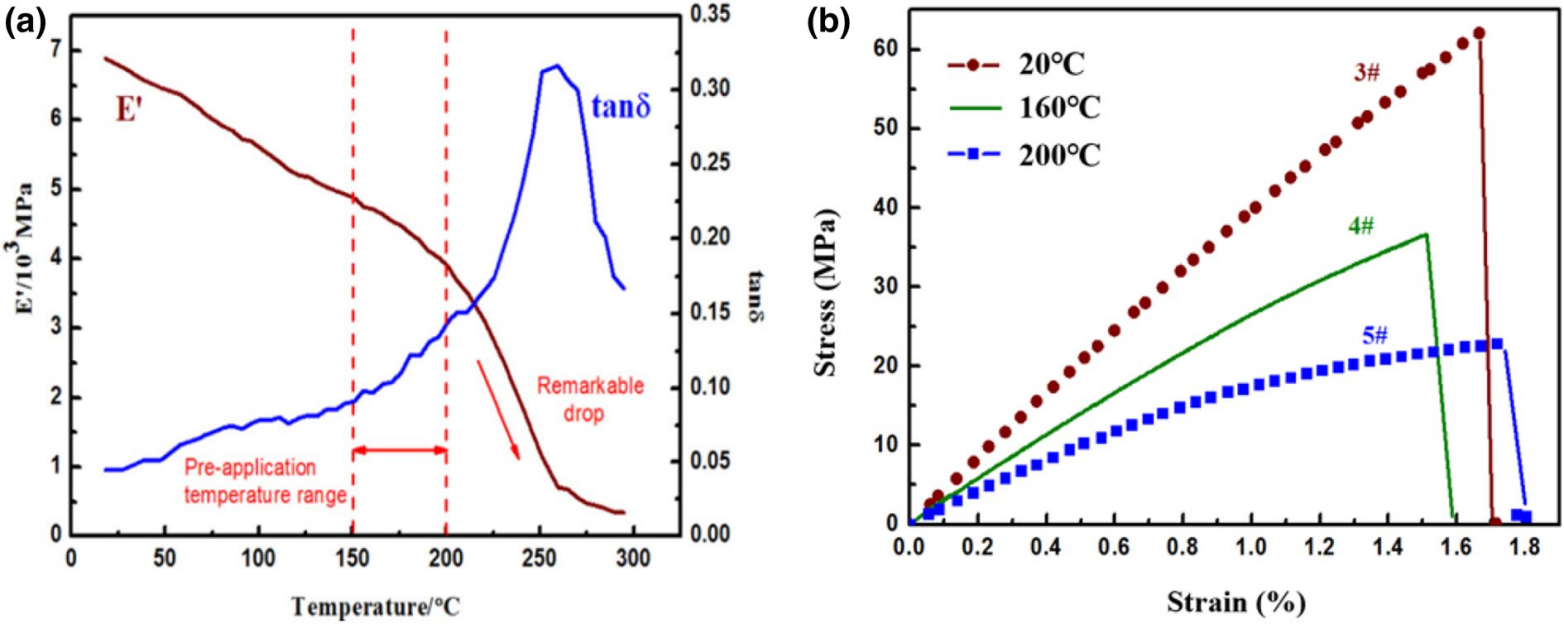
(**a**) DMA curve of QY8911-IV resin. (**b**) Tensile stress–strain curves of QY8911-IV resin at different temperature.

It is well known that resin matrix plays a significant role in the tensile responses and failure modes of fiber reinforcement composites at different temperature^15,38^. As shown in Fig. 1b,c, resin exists in the resin-rich domain and inside of fiber yarns. Figure 5 illustrates the dynamic mechanical analysis (DMA) curve of QY8911-IV resin and tensile stress–strain curves of CF/QY8911-IV [90]_8_ at 20 °C, 160 °C and 200 °C. The dimensions of samples for DMA and tensile tests are 15 mm × 15 mm × 5 mm and 300 mm × 25 mm × 1.59 mm, respectively. The aforesaid dimensions have been added in the revised manuscript It can be seen from Fig. 5a that the storage modulus, E’, declines with the increase of temperature from 20 °C to 200 °C, but it experiences a rapid drop, indicating that the mechanical properties of resin will obviously reduce after 200 °C. Furthermore, the glass transition (*T*_g_) of ~ 256 °C is seen as a peak value in the energy dissipation, tan δ, vs. temperature curve. In addition, referring to the stress vs. strain curves (Fig. 5b), the tensile modulus and strength retention of QY8911-IV resin decline significantly at 160 °C. When the temperature rises to 200 °C, the modulus and strength retention experience a more severe declination. As listed in Table 2, the modulus and strength retention rates at 160 °C are 69.23% and 57.21%, and those at 200 °C only remain 48.80% and 34.05%. Compared to the corresponding data given in Table 1, the higher retention rates can be associated to the 3D net-shaped architecture joined together by fiber yarns.

**Table 2:** Mechanical properties of QY8911-IV at various temperatures.

No	Temperature (°C)	Modulus (GPa)	Average (GPa)	Strength (MPa)	Average (MPa)
1	20	3.95	4.16	49.51	68.28
2		4.52		63.86	
3		4.02		79.46	
4	160	2.84	2.88	37.84	39.06
5		2.92		36.30	
6		–-		43.03	
7	200	1.98	2.03	22.18	23.25
8		1.93		25.62	
9		2.17		21.96	

**Figure 6:**
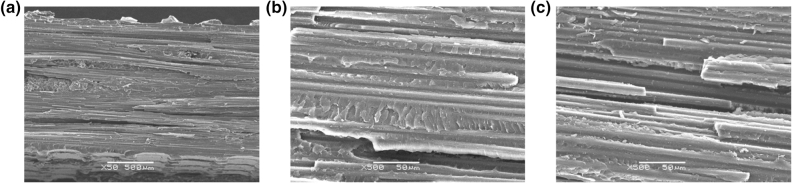
SEM micrographs of resin matrix at different temperatures: (**a**) 20 °C; (**b**) 160 °C; (**c**) 200 °C.

Apart from the effect of temperature on the mechanical properties of resin, the bonding performance between fiber and matrix should be considered at elevated temperatures. The fracture morphologies as demonstrated in Fig. 6 suggest the bonding states of CF/QY8911-IV [90]_8_ unidirectional composites at various temperatures. As illustrated in Fig. 6a,b, large-scale matrix hackles are found along the fibers at 20 °C and 160 °C, and substantial fibers are completely covered by the thick matrix layer. It is suggested that the resin matrix remains a brittle characteristic at 20 °C and 160 °C, and the fiber and matrix is well bonded. However, at 200 °C, few resin adheres to fibers, and plastic hackles are noted, indicating that the softening of matrix at a large degree and possesses a weak interface bonding. It can be concluded from the viewpoint of resin matrix that for the 2.5DWC with/without a center hole, the softening of matrix at 180 °C in the resin-rich domain results in the rotation of load-carrying fiber yarns towards the loading direction, enhancing the load-bearing capacity to some degree. But, at 200 °C, excessive high temperature leads to massive failure of matrix and interface, and therefore the strength retention rates obvious drop (Fig. 4).

### Effect of temperature on fracture morphologies of 2.5DWC without a center hole

**Figure 7:**
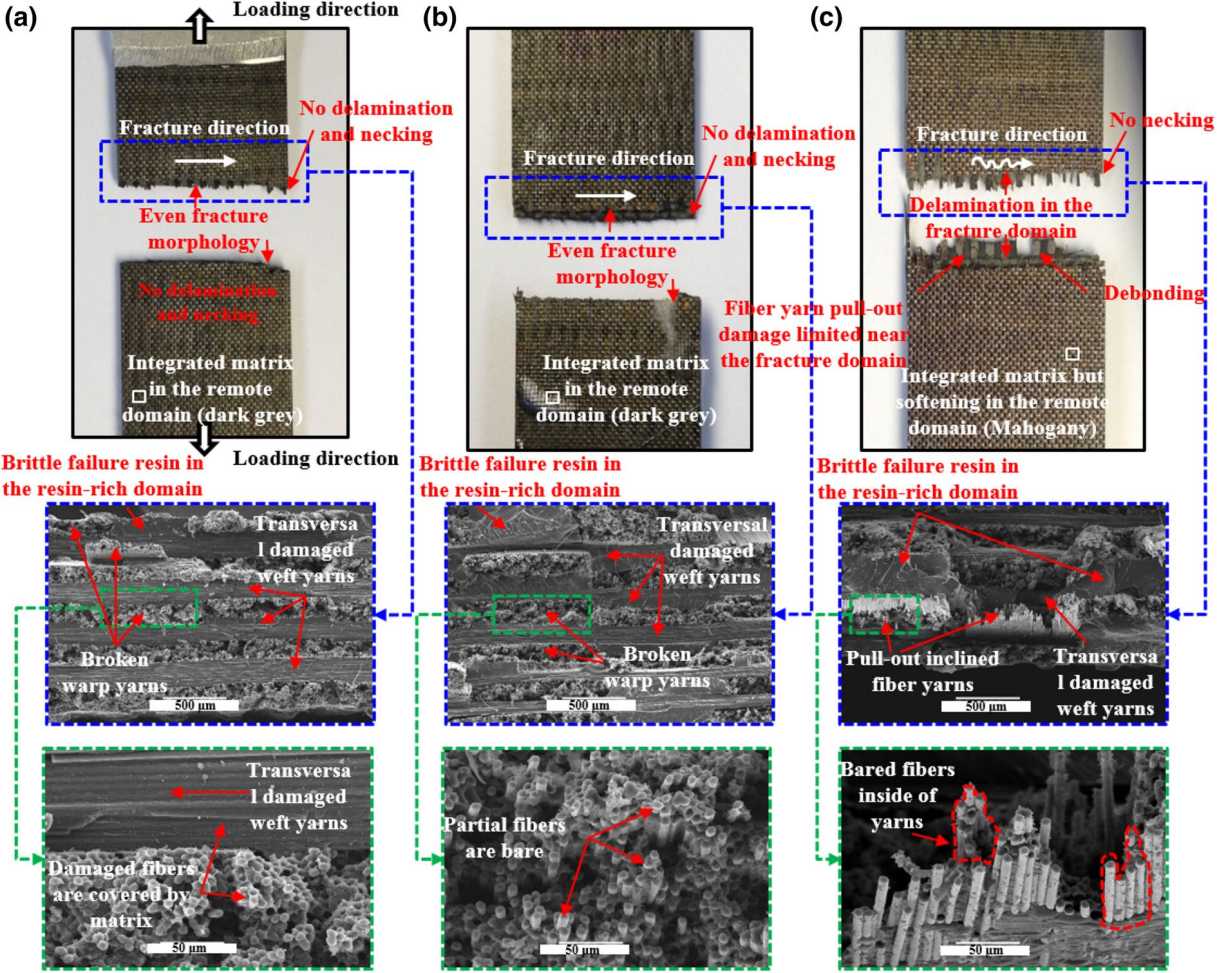
Macroscopic and microscopic images of the failed 2.5DWC without a center hole at different temperatures. (**a**) 20 °C; (**b**) 180 °C; (**c**) 240 °C.

To be the best of our knowledge, the tensile behavior and failure modes greatly depend upon the experimental temperature for the samples with a center hole. Figure 7 presents the corresponding fracture morphologies at different temperatures.

At 20 °C, the macroscopic fracture morphology is considerably even, and the crack propagation direction is totally perpendicular to the loading direction, viz. weft direction. It should be noted that there is nearly no delamination and necking phenomenon near the fracture site, which is similar with the fracture morphology of unidirectional composites^15^, indicating that the 2.5DWC has a good structural integration. In addition, from the microscopic SEM images, inclined warp yarns in the failed cross-section are all broken, suggesting that they are the principal load-bearing elements. The fiber-dominated failure mode was also noted for other 3D woven composites^39,40^. Moreover, the transversal damaged weft yarns and brittle failed resin in the resin-rich domain are found, but fibers inside of fiber yarns are still covered by lots of resin.

Figure 7b illustrates that the fracture morphology at 180 °C is basically similar with that at 20 °C. First, there is no obvious delamination damage phenomenon near the fracture surface for each case. Second, both the fracture surfaces are quite even, and no necking phenomenon is noted. Third, the corresponding fracture direction is perpendicular to loading direction, giving an indication of a uniform load-bearing capacity. It indicates that the samples still possess a high load-bearing capacity at 180 °C, which is well agreement with the experimental data shown in Table 1. From the microscopic view, substantial broken inclined warp yarns in the cross-section are still found, alongside of transversal damaged weft yarns and brittle failed resin in the resin-rich domain. But, particularly, filament bundles in fractured carbon tow are bare, meaning that the interfaces among fibers are to some degree affected by temperature. At 240 °C, the color of matrix in the remote domain has turned from dark grey to mahogany (Fig. 7c), suggesting that resin becomes soften seriously. The fracture surface is no longer uniform, and delamination as well as pull-out damaged fiber yarns are clearly observed. But, there is still no necking phenomenon and the fracture site is totally restricted within the warp direction length of smallest repeating unit (Fig. 1c). From the SEM images, the inclined warp yarns in the cross-section are also broken, and massive broom-like broken fibers are found inside of fiber yarns. Apart from the failed resin, the transversal damaged weft yarns still exist in the cross-section. Moreover, the fibers inside of broken warp yarns are bare, verifying that the temperature plays an important role in the mechanical properties of resin and interfaces.

### Effect of temperature on fracture morphologies of open-hole 2.5DWC

**Figure 8:**
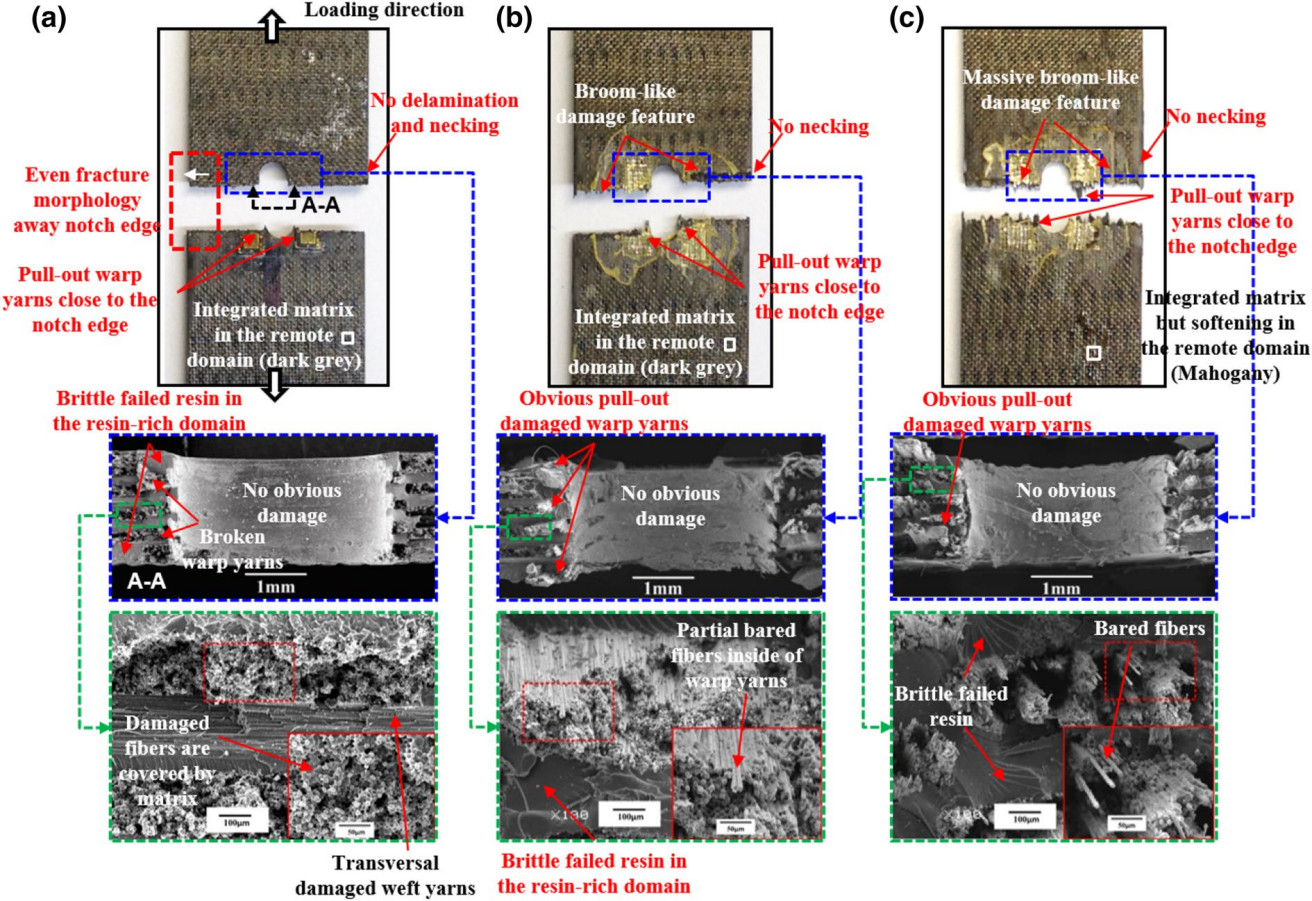
Macroscopic and microscopic images of the failed 2.5DWC with a hole size of 4.1 mm at different temperatures. (**a**) 20 °C; (**b**) 180 °C; (**c**) 240 °C.

**Figure 9:**
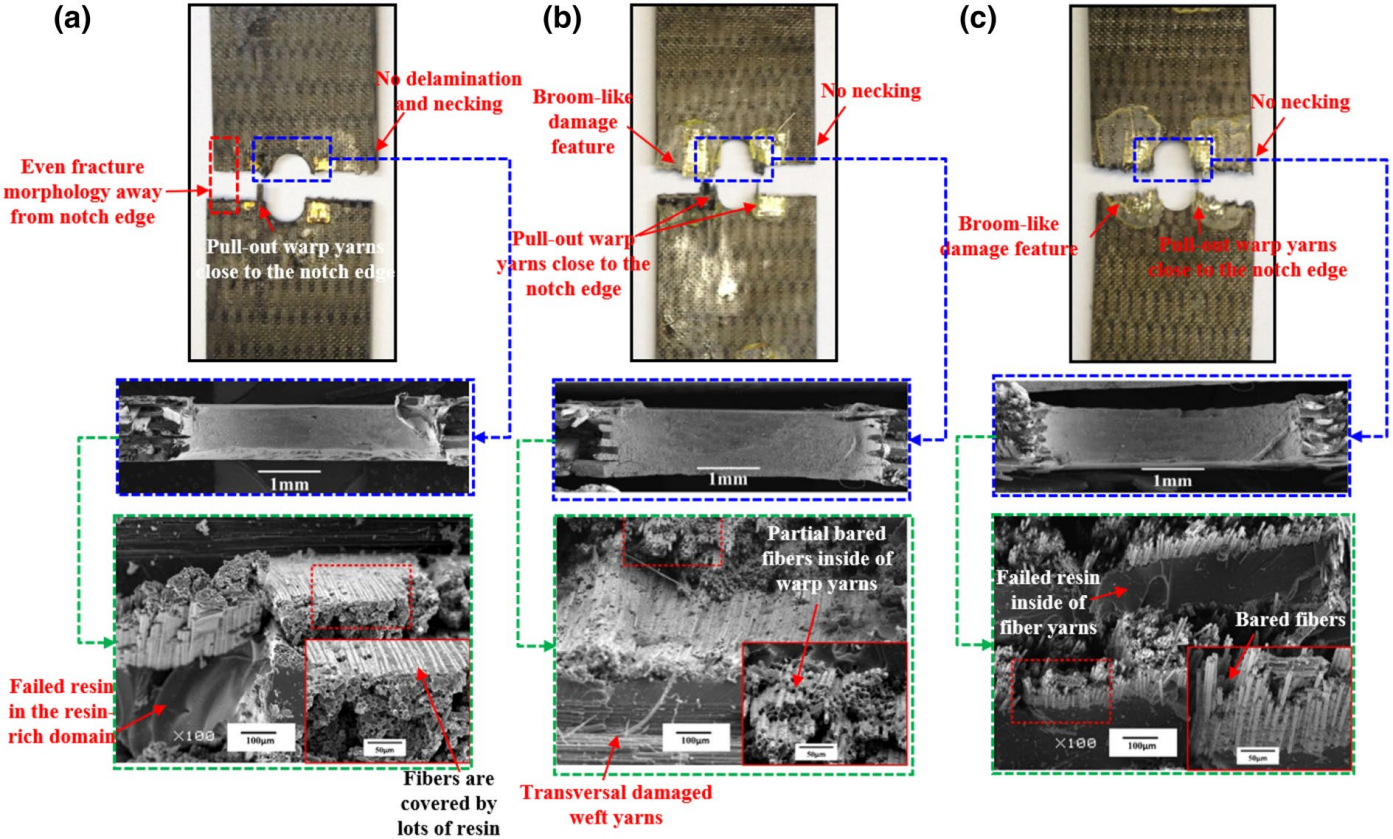
Macroscopic and microscopic images of the failed 2.5DWC with a hole size of 6.3 mm at different temperatures. (**a**) 20 °C; (**b**) 180 °C; (**c**) 240 °C.

**Figure 10:**
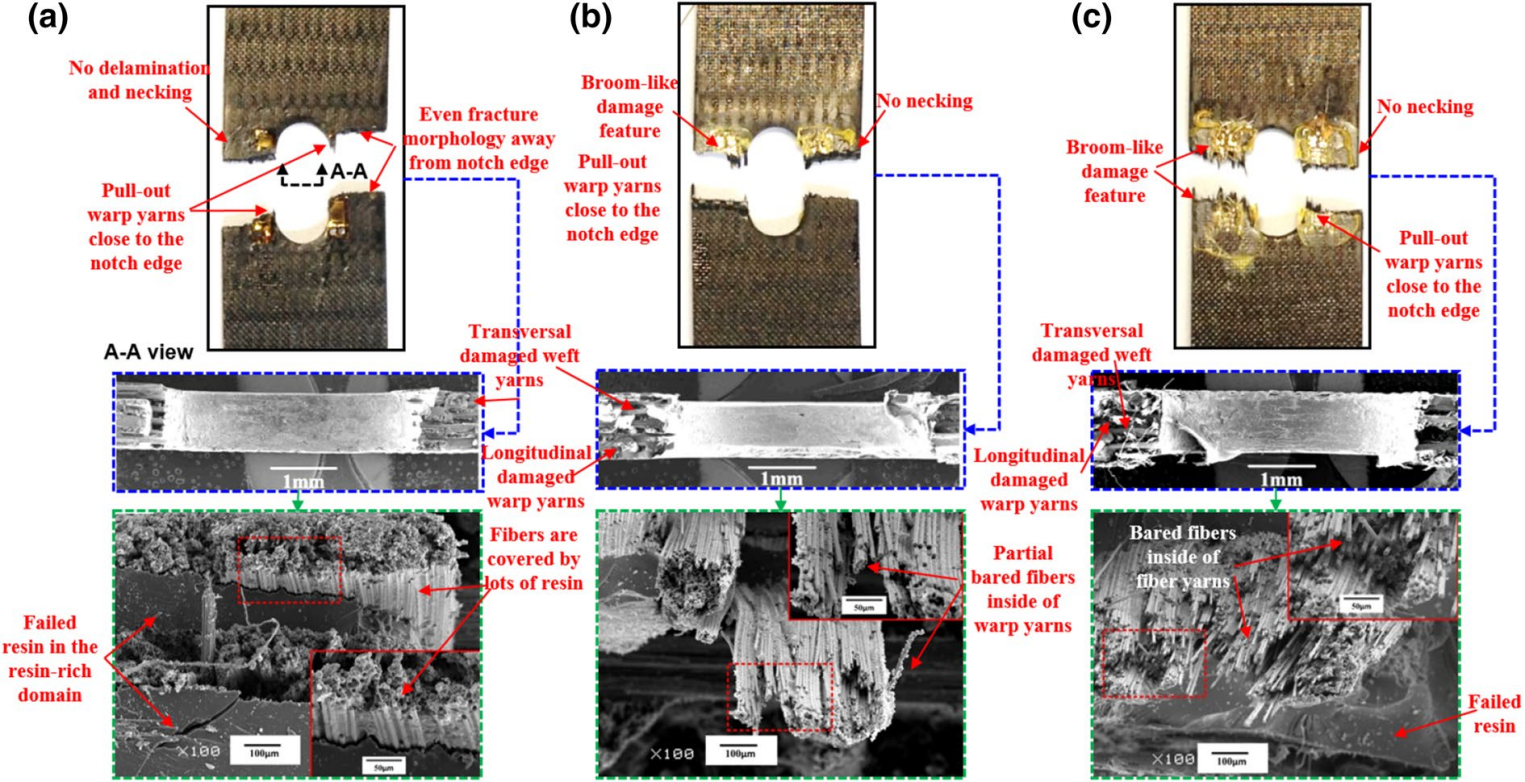
Macroscopic and microscopic images of the failed 2.5DWC with a hole size of 8.5 mm at different temperatures. (**a**) 20 °C; (**b**) 180 °C; (**c**) 240 °C.

Figures 8, 9, 10 illustrate the fracture morphologies of the open-hole 2.5DWC at 20 °C, 180 °C and 240 °C. Previous researches in terms of open-hole laminated/2D woven composites^8,18,41–44^ have shown that the large-area delamination fracture morphologies can be examined near the hole-edge domain, particularly at elevated temperatures. In this work, for the samples with a hole size of 4.1 mm, due to the occurrence of z-direction interlinkage (Fig. 1b,c), no obvious large-area delamination and necking phenomena are observed in the cross-section at 20 °C, 180 °C and 240 °C (Fig. 8), which also confirms that the fracture mechanism is dominated by the 3D net-shaped carbon fiber yarns. More specifically, from the macroscopic images, the failed cross-section at 20 °C shown in Fig. 8a exhibits a considerably even fracture morphology, but pull-out warp yarns are observed near the hole edge, which could be ascribed to the effect of hole-edge stress concentration. When increasing the temperature, more broom-like damage features are inspected, indicating that the interfaces of fiber yarns/yarns and fiber/matrix are remarkably affected by temperature. However, near the hole edge, there are still pull-out damaged warp yarns, and this phenomenon was also found by the Ref.^32,45^. The fracture sites at 180 °C and 240 °C are also restricted within a warp direction length. From the microscopic view, longitudinal warp yarn damage mode, transversal weft yarn damage mode and resin failure in the resin-rich domain are found in the cross-section. Moreover, all the inclined warp yarns are broken on the root. From the enlarged SEM images, plastic strips are observed among the fiber yarns, giving a suggesting of the brittle failure for resin in the resin-rich domain. And more and more bared fibers inside of fiber yarns are noted, revealing the resin inside of fiber yarns fails, giving rise to the weakness of interface performance.

For the samples with a hole size of 6.3 mm and 8.5 mm, shown in Figs. 9 and 10, the pull-out warp yarns near the hole edge and broom-like damage features without necking phenomenon are also observed at 20 °C, 180 °C and 240 °C, which are very similar with that of samples with a hole size of 4.1 mm. From the microscopic images, all the inclined warp yarns are broken on the root, and transversal damaged weft yarns as well as failed resin are also found around the broken warp yarns. Additionally, the number of bared fibers is also significantly increased as the rise of temperature. Thus, based on the aforesaid inspections, the influence of temperature on the fracture morphologies of open-hole 2.5DWC is more obvious than that of 2.5DWC without a center hole.

Compared to the fracture morphologies of samples without a center hole at the same temperatures, the fracture morphologies of open-hole ones are similar, apart from the hole-edge domain, which suggests that the effect of hole on the tensile behaviors is restricted within the hole-edge domain due to the 3D net-shaped architecture. It is very meaningful for the material to apply in the composite structures. However, the pull-out damaged warp yarns and later discussed strain behaviors (“Initial equivalent stress distribution”) are verified the hole-edge stress concentration.

### Initial equivalent stress distribution

**Figure 11:**
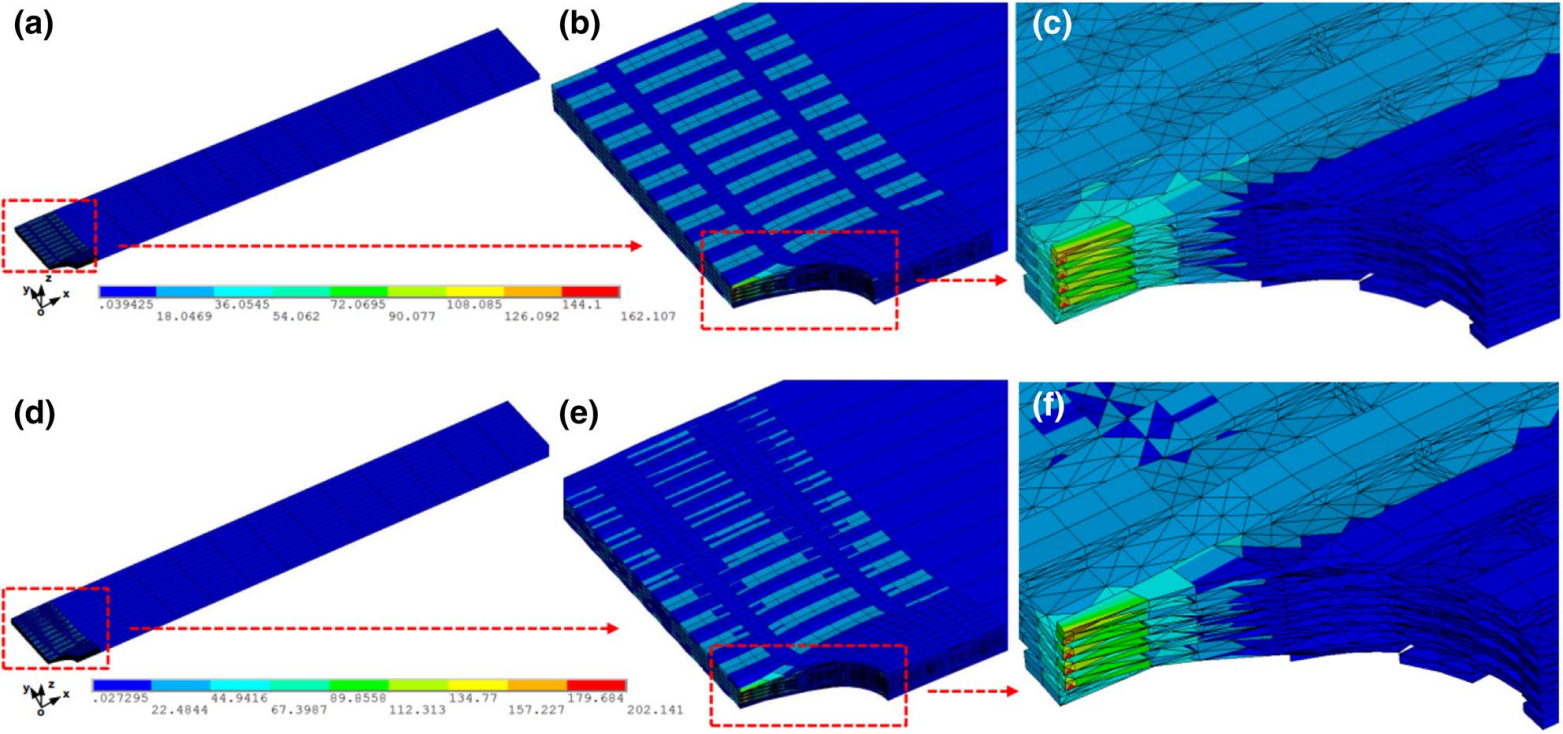
Initial deformed von Misses stress contours of FEM. (**a**–**c**) entire, magnification and local stress contours simulated at 20 °C. (**d**–**f**) entire, magnification and local stress contours simulated at 180 °C.

Figure 11 exhibits the initial equivalent stress distribution of open-hole 2.5D woven composites subjected to warp direction loading at 20 °C and 180 °C (“X” axis in Fig. 11, initial pressure loaded on the end surface of samples is 0.1 MPa). It can be clearly seen that the stress distribution is not uniform. To be more exactly, the hole-edge regions emerge obvious stress concentration in comparison with the remote regions, especially the hole-edge warps component, the maximum stress of which is approximately 5 times greater than that of the remote regions (Fig. 11c,f). Moreover, the stress in warps (not to be considered the cut-off yarns) is also greater than that in the others, indicating that the warps are the primary load-carrying objects when it comes to warp loading, regardless of temperature condition (Fig. 11b,e). Likewise, due to the obstruction of consecutive warp yarns, stress concentration phenomenon is effectively restricted in a small region (about one warp’s width along the “Y” axis direction) rather than large-area stress concentration likely in notched laminated/2D composites ^43^.

In addition to stress distribution, the higher stress state at 180 °C is adequately suggestive of the influence of thermal stress induced by considerable discrepancy between fiber and resin (Fig. 11a,d). The warps’ stress distribution along the “Y” axis appears a certain degree of gradient distribution characterization, which could be attributed to the effect of thermal stress, but the warps still undertake the main loading.

### Localized strain responses at various temperatures

**Figure 12:**
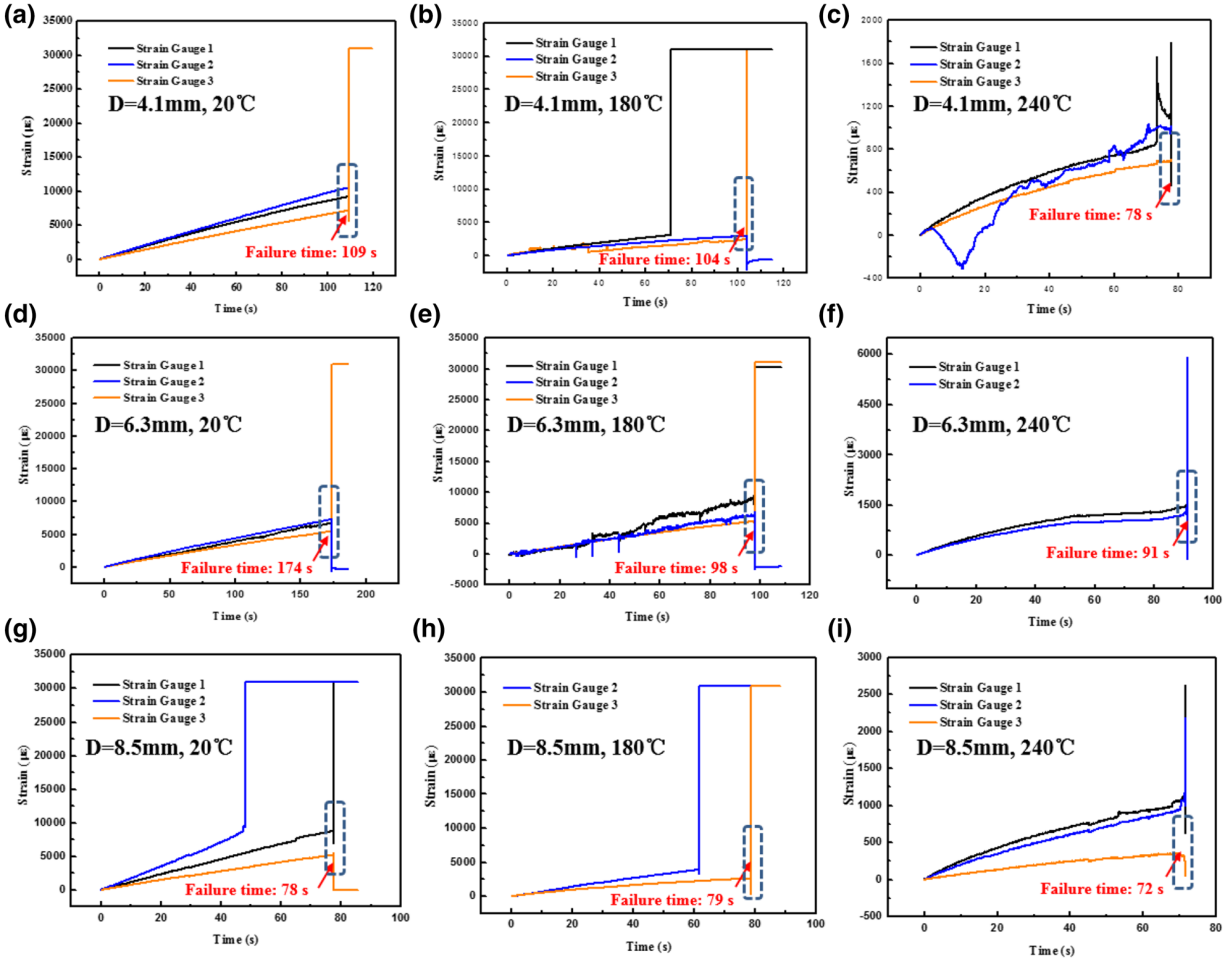
Strain vs. time curves obtained from the strain gauges of open-hole 2.5DWC at various temperatures.

Figure 12 presents the localized strain vs. time curves obtained from the three strain gauges adhered to three signed sites (Fig. 1a). Here, the data onto the curves present a certain degree of discreteness and premature failure of some strain gauges, especially at elevated temperatures (Fig. 12b,c,e,g,h). But, in general, the real-time strains measured by the strain gauges “1” and “2” are quite similar (Fig. 12a,c,d,f), which are basically higher than those measured by the strain gauge “3” (adhered to the remote domain). It indicates that the stress concentration phenomenon still exists near the hole-edge domain for the open-hole 2.5DWC. When temperature remains constant, there is a linear relationship between the strain and time at 20 °C, which also confirms that the open-hole 2.5DWC possesses a great structural integration, thereby resulting in a brittle fracture characteristic. However, a nonlinear behavior in the strain- time curves is found at 180 °C or 240 °C, indicating that the effect of temperature on the mechanical behaviors. Interestingly, in most cases, even though the discrepancies indeed exist at various temperatures, the failure time points are basically same for the strain gauges measured adhered to the hole-edge sites and the remote site (Fig. 12). Additionally, it can be seen that the failure time gradually reduces with the increase of temperature for the samples with the same hole size. It indicates that the load-carrying capacity of open-hole 2.5DWC gradually weakens with the rise of temperature. Thus, it gives an indication of hole-edge stress concentration, regardless of temperature.

## Discussion

**Figure 13:**
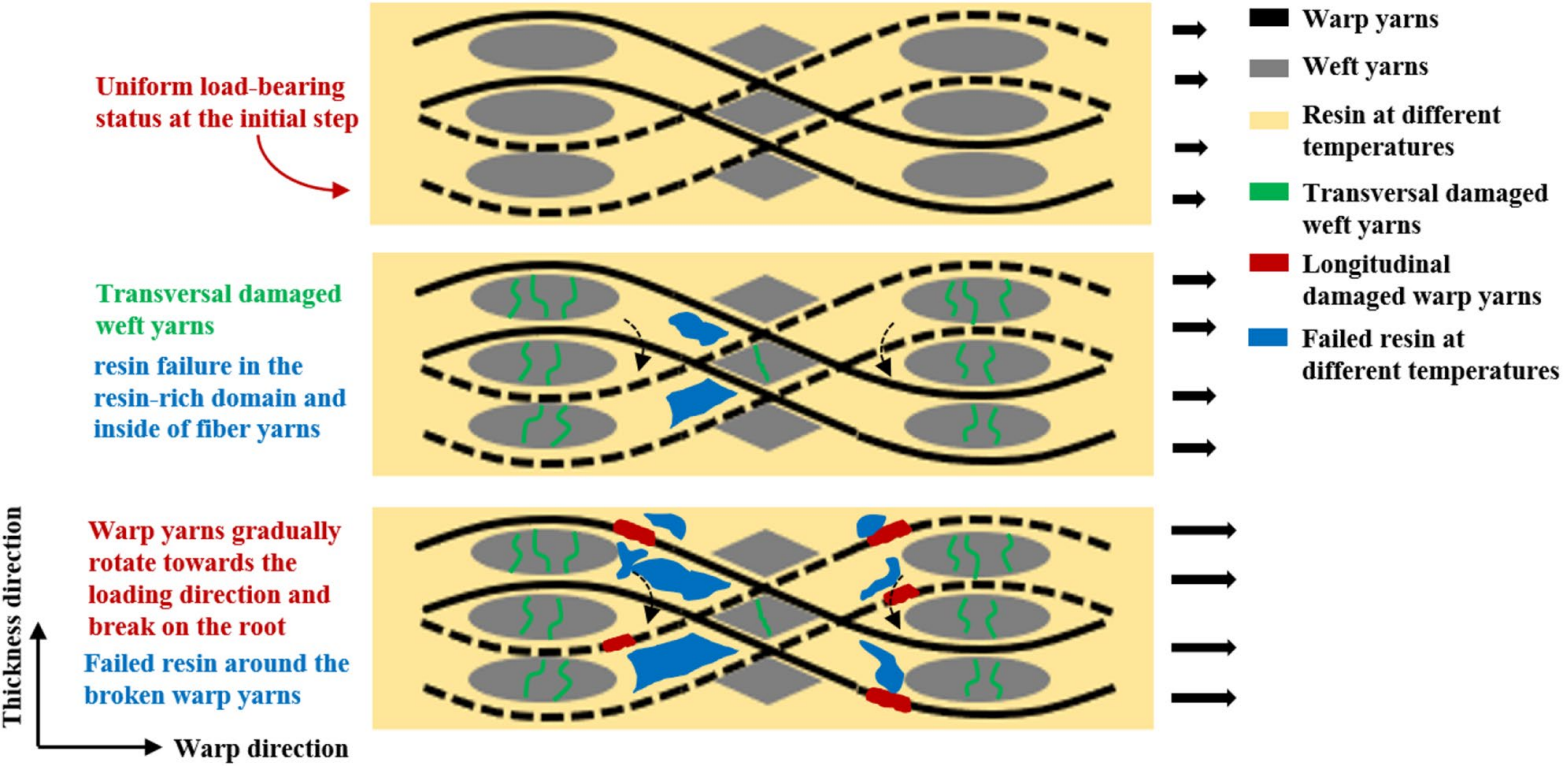
Damage mechanisms of 2.5DWC without a center hole at different temperatures.

**Figure 14:**
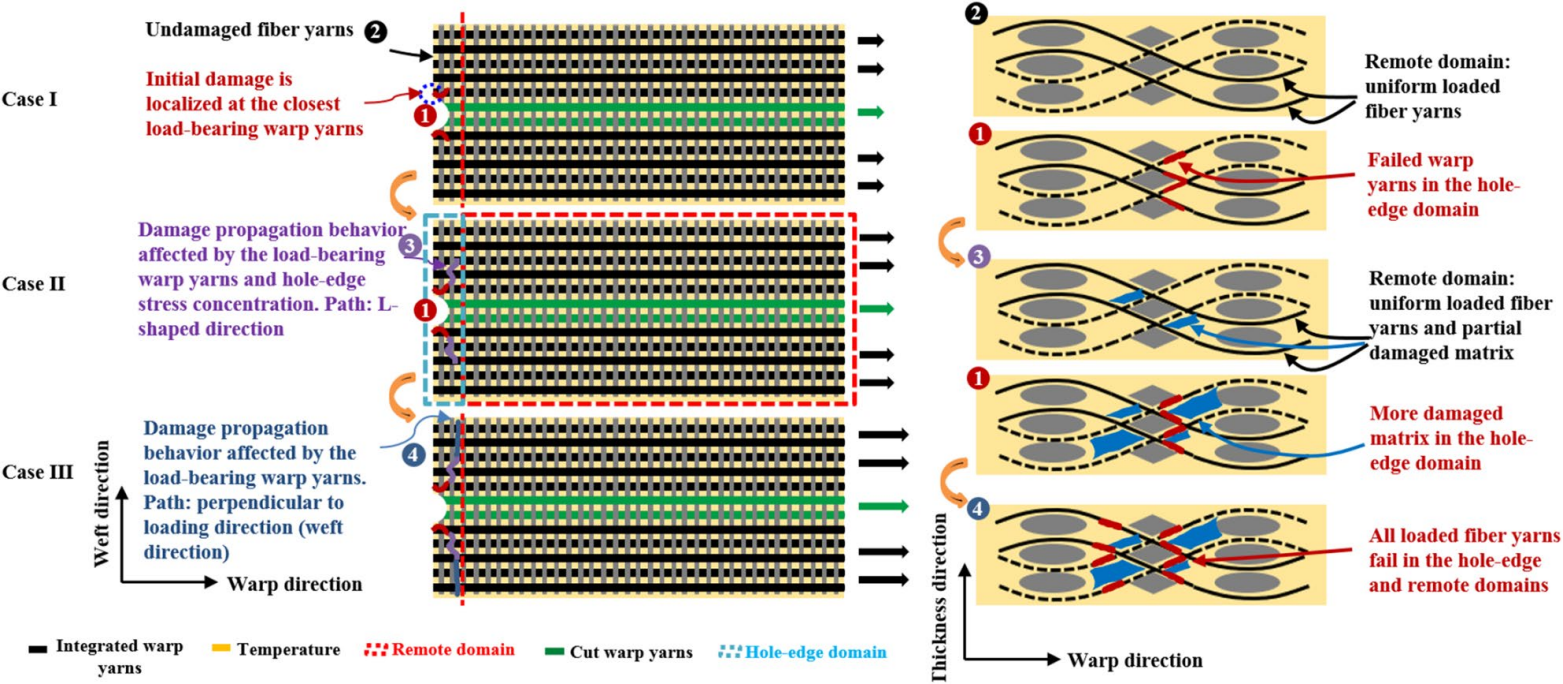
Damage mechanisms of open-hole 2.5DWC at different temperatures.

As the mentioned above, the fracture mechanisms of 2.5DWC with/without a center hole may be relevant with the load-bearing fiber yarns, hole and temperature. Figures 13 and 14 show the corresponding probable fracture mechanisms. For the samples without a center hole, uniform load-bearing status results in a linear response at the initial step. As the increase of load along the warp direction, matrix in the resin-rich domain and inside of fiber yarns fails (Fig. 13i,ii), which can be attributed to the squeeze of load-bearing warp yarns. In accordance with the stress–strain curves shown in Fig. 2, owing to the 3D net-shaped architecture, the aforesaid matrix cracking has a weak effect on the mechanical properties of samples without a center hole. Once the principal load-carrying warp yarns break, a brittle fracture behavior will be activated, simultaneously further giving rise to resin failure and transversal yarn damage (Fig. 13iii). When samples are loaded at a relatively high temperature, on one hand, it is beneficial to accommodate the stress concentration among various components, due to the softening of resin. On the other hand, the softened resin to some degree loses the support capacity, causing that warp yarns have a more uniform load-bearing status. Therefore, the fracture morphology of samples without a center hole at 180 °C remains even and no delamination (Fig. 7b). However, at 240 °C, the mechanical properties of resin and interface bonding capacity seriously drop, and the enhancing effect by the aforementioned analysis is weaker than the damage effect. Ultimately, the warp yarns thoroughly fail and then the composites lose the bear-carrying capacity, in which obvious resin failure and interfacial debonding phenomena were observed in the cross-section (Fig. 7c).

For the open-hole samples, the hole-edge domain has a higher stress level (Figs. 11 and 12), causing that the initial cracks, involving the resin failure and transversal damaged weft yarn, emerge near the hole edge (Fig. 14i). The warp yarns closest to the hole edge firstly happen breakage, and cracks in terms of longitudinal yarn damage, transversal yarn damage and resin failure modes propagate along the broken warp yarns (Fig. 14ii). As the cracks propagate away from the hole edge, the effect of hole-edge stress concentration gradually is weakened and composites can take the warp direction load uniformly. In the following process, the crack propagation path is totally perpendicular to warp direction, therefore forming a “*L*” damage propagation path (Fig. 14iii). When increasing the temperature, the hole-edge stress concentration can be effectively accommodated due to the softening of resin (Fig. 5). More obvious broom-like fracture morphologies were noted in the cross-section, which includes that resin failure and interfacial debonding. At 180 °C, the softening of resin would result in a larger load-bearing capacity of integrated warp yarns, which is very similar with that for the samples without a center hole. Due to the aforementioned two reasons, the strength retention rates of open-hole 2.5DWC maintain a relatively high level (Fig. 4).

## Conclusion

The effects of temperature on the mechanical properties and fracture morphologies of 2.5D CF/QY8911-IV woven composites with/without a center hole under tension were experimentally studied in this work. Some meaningful concludes have been obtained, particularly at elevated temperatures.

1. All the stress–strain curves of 2.5DWC without a center hole present a linear response, followed by a slightly nonlinear at the end of curve. Brittle failure characteristic was found especially at elevated temperatures (180 °C and 240 °C). Combined with the DMA curve of resin, the sudden failure phenomenon indicates a fiber-dominated failure mechanism for the 2.5DWC without a center hole at different temperatures. The strength retention rates of open-hole 2.5DWC at 180 °C are higher than those of samples without a center hole, which can be attributed to two aspects: ① the accommodation of stress concentration at 180 °C; ② the rotation of load-bearing warp yarns towards loading direction. However, the strengths of open-hole samples with various hole sizes remarkably decrease when the temperature rises to 240 °C.
2. Fracture morphologies of 2.5DWC with/without a center hole at different temperatures were inspected from the macroscopic and microscopic views. Besides of the hole-edge domain, the fracture morphologies of open-hole samples are very similar with those of samples without a center hole. No delamination and necking damage modes of samples with/without a center hole in the cross-section below 180 °C, but a broom-like failure characteristic becomes obvious with the increase of temperature, owing to the effect of temperature. However, in the hole-edge domain, a “*L*” shaped damage mode was found, which can be ascribed to the comprehensive effects of hole-edge stress concentration and primary load-bearing warp yarns. From the macroscopic views, the damage modes include longitudinal warp yarn damage, transversal weft yarn damage and resin failure. From the microscopic views, although the fracture morphologies of samples are still fiber-dominated failure, more and more bared fibers inside of yarns were observed at 240 °C, suggesting that the temperature has an important impact on the interfacial performance.
3. Referring to the test data and fracture morphologies, probable damage mechanisms of 2.5DWC with/without a center hole at different temperatures were proposed. The crack propagation paths were significantly affected by the principle load-bearing warp yarns. In the loading process, the cracks in terms of transversal weft yarn damage and resin failure modes initially occur, and the warp yarns would rotate towards the loading direction, resulting in the longitudinal warp yarn damage mode. Moreover, resin matrix plays an important role in the damage modes of 2.5DWC with/without a center hole, particularly at elevated temperatures. On one hand, the mechanical properties of resin significantly decreased with the increase of temperature, confirmed by DMA curve. On the other hand, the interfaces among fiber yarns/yarns and fiber/matrix were obviously affected by temperature, demonstrated by broom-like fracture morphology. For the open-hole samples, the damage propagation paths in the remote domain are well consistent with those of samples without a center hole. But in the hole-edge domain, the stress distribution is not uniform. Therefore, it caused that the warp yarns closest to hole edge firstly failed and a “*L*” shaped fracture morphology was observed.

## Supplementary information

Supplementary information
