# Supplementary material for: Tensile behaviors of layer-to-layer 2.5D angle-interlock woven composites with/without a center hole at various temperatures
Source: Sci Rep. 2020 Sep 18;10:15332. doi: 10.1038/s41598-020-71481-1 (PMC7501272; doi:10.1038/s41598-020-71481-1)
Supplement: Supplementary file 1 — Supplementary information [file 41598_2020_71481_MOESM1_ESM.docx]

**Supporting Information (SI)**

# Tensile behaviors of layer-to-layer 2.5D angle-interlock woven composites with/ without a center hole at various temperatures

Jian Song^a^*, Weidong WEN^b^, Haitao Cui^b^, Lixiao Li^a^, Yang Lu^c^*

^a^ Guangdong Provincial Key Laboratory of Durability for Marine Civil Engineering, College of Civil and Transportation Engineering, Shenzhen University, Shenzhen 518060, China

^b^ College of Energy and Power Engineering, Nanjing University of Aeronautics and Astronautics, Jiangsu 210016, China

^c^ Nano-Manufacturing Laboratory (NML), Shenzhen Research Institute of City University of Hong Kong, Shenzhen, 518057, China

*Corresponding author: *Jian Song* ([jiansong@szu.edu.cn](mailto:jiansong@szu.edu.cn)), *Yang Lu* ([yanglu@cityu.edu.hk](mailto:yanglu@cityu.edu.hk))

**S. 1 Finite element analysis**

**S. 1. 1 Establishment of finite element model**

Due to existence the periodical characterization of 2.5D woven composites, a FE model without circle notch was first established. Based on the actual microstructure (**Fig.1b~c**, ***Manuscript***), two assumptions were proposed: (i) A rectangular and two anti-quadratic curve shapes are selected to describe the cross-sections of warps and wefts respectively; (ii) The wefts are assumed to be straight, but the trend of warp is composed of two parts (“AC” and “CD” sections in **Fig. S1b**), viz a quadratic curve depending on the configuration of weft and a straight line reflecting the tightening effect in the woven process. The inner representative cell, named Inner-cell model, can be fulfilled as following process.

- The boundary dimensions of the inner cell

$L_{x}=10(N_{f}-1)/M_{w},L_{y}=10N_{j}/M_{j}$ (S1)

where $L_{x}$and $L_{x}$are the warp direction (“X” axis in **Fig. S1a**) and weft direction (“Y” axis in **Fig. S1a**) length (mm) respectively; $N_{f}$ is the number of wefts at the same height (“Z” axis in **Fig. S1a**); and $M_{j}$and $M_{w}$are the warp and weft arrange density.

- The cross-sectional sizes of the warp

$A_{j}=\frac{T}{1000\rho P_{j}},W_{1j}=\frac{10}{M_{j}},W_{2j}=\frac{A_{j}}{W_{1j}}$ (S2)

where $A_{j}$is the cross-sectional area of warp ($mm^{2}$);$T$is the linear density of yarns ($g/1000m$); $\rho$is the material density ($g/cm^{3}$);$P_{j}$ is the fiber aggregation density.

- Other independent parameters and Full-cell model

Based on the aforementioned assumption, the configuration of weft is assumed quadratic curve (“AC” section in **Fig. S1b**, viz.$z=ax^{2}+bx+c$. Additionally, due to the continuity and first-order continuity conditions in point “C” as well as geometric relationship in “CD” section (**Fig. S1b**), following equations can be obtained.

$\frac{W_{2j}+W_{2w}}{2}=a\left(\frac{W_{1w}}{2}\right)^{2}+b\left(\frac{W_{1w}}{2}\right)+c$ (S3)

$\left.z^{'}\right|_{x=W_{1w}/2}=-\tan\theta=2a\frac{W_{1w}}{2}$ (S4)

$\tan\theta=\frac{W_{2j}+W_{2w}+W{}_{2j}\cos\theta}{L_{x}/2-W_{1w}-W_{2j}\sin\theta}$ (S5)

where$\theta$is the inclination angle of warps. By making sure the area of weft is equal to$A_{w}$, that is:

$4\int_{0}^{W_{1w}/2} \left(ax2+bx+c-\frac{W_{2j}+W_{2w}}{2}\right)dx=A_{w}$ (S6)

By solving **Eqs. (S3)~(S6)**, the independent parameters ($W_{1w}$, $a$, $c$ and $\theta$) can be calculated, and the inner-cell model can be established. Afterwards, taking the effect of RTM technology (**Fig. S1c**) into consideration, representative volume cell, named Full-cell model, can be established.

- Open-hole 2.5D woven composites

Owing to the repetitive characterization and calculation efficiency of 2.5D woven composites, only 1/4 model was used to simulation. As for the notch-edge region, taking the length of one Full-cell model as the length of notch-edge refine region, several Full-cell models were established, where the number of Full-cell along the “Y” axis is dependent on the half width of sample. As for the remote region, taking the length as well as width of one Full-cell model as the corresponding sizes of equivalent hexahedron, the residual region, viz remote region, can be built up, where the amount of hexahedron along the “X” axis is also dependent on the length of sample. Hence, the open-hole 2.5D woven composites can be fully established, as shown in **Fig. S1d**.


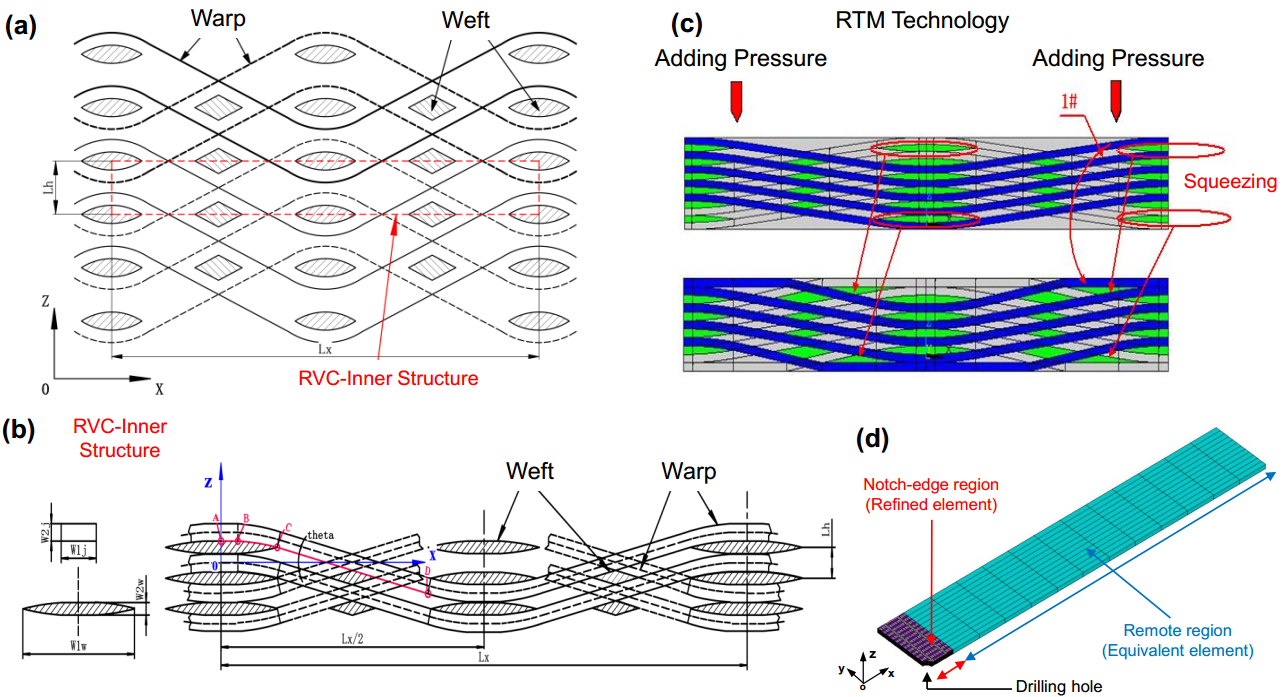


**Fig. S1** Establishment process of open-hole 2.5D woven composites. (a) Schematic diagram of woven configuration. (b) Geometric relationship without the outer layer structure. (c) RTM process. (d) FE modeling of open-hole 2.5D woven composites.

**S. 2 Load versus displacement curves of open-hole 2.5D woven composites**


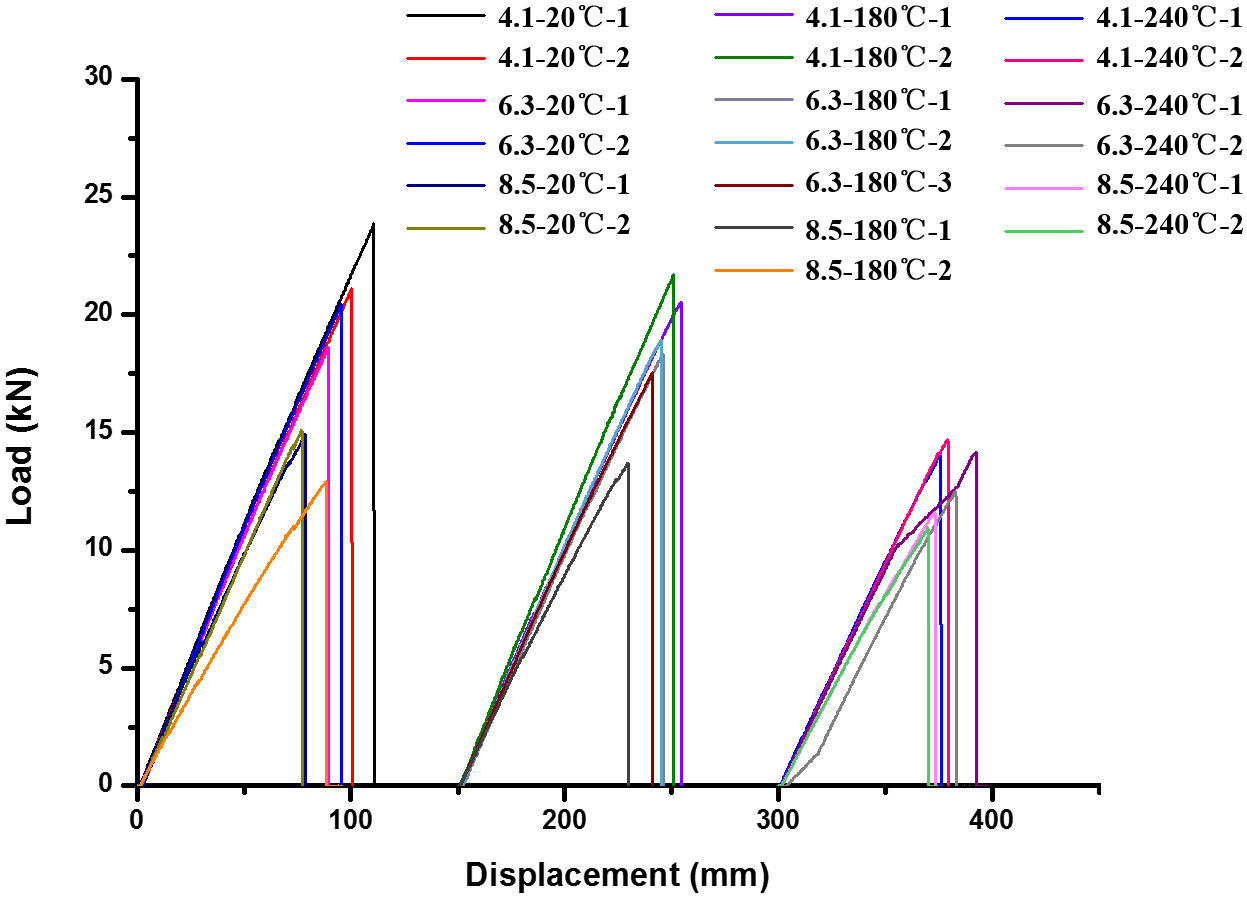


**Fig. S2** All the load vs. displacement curves of open-hole 2.5DWC at different temperatures.

**S. 3 Coupling effect of temperature and hole size**


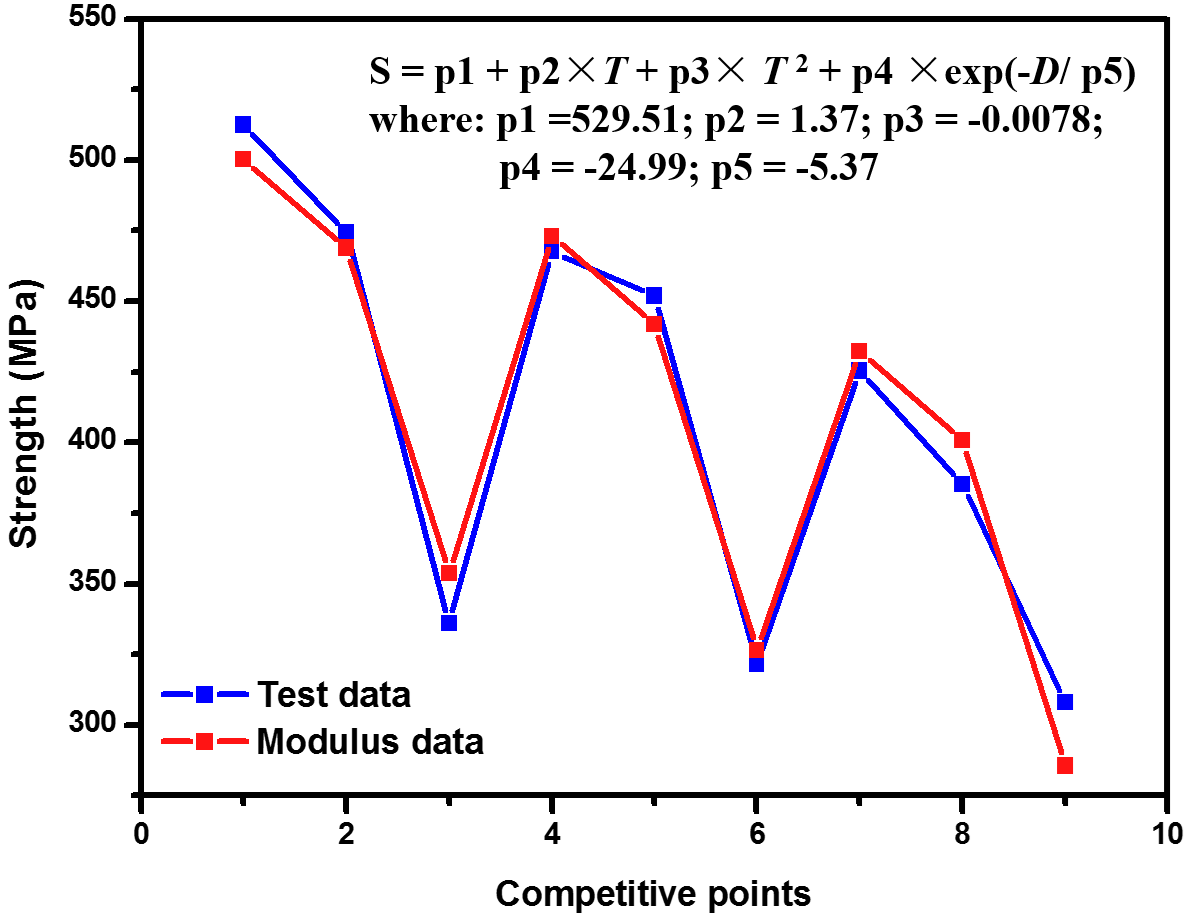


**Fig. S3** Tensile strength of open-hole 2.5DWC as a function of temperature and hole size.

The relationship between strength and temperature and hole size can be quantitatively expressed as:

$S=529.51+1.37\times T-0.0078\times T^{2}-24.99\exp(D/5.37)$ (S7)

where *S*, *T* and *D* indicate the tensile strength, temperature and hole dimeter, respectively.
